# Hospitalized Older Patients with *Clostridioides difficile* Infection Refractory to Conventional Antibiotic Therapy Benefit from Fecal Microbiota Transplant

**DOI:** 10.20900/agmr20210012

**Published:** 2021-04-30

**Authors:** Jae Hyun Shin, Rachel Ann Hays, Cirle Alcantara Warren

**Affiliations:** 1 Division of Infectious Diseases and International Health, Department of Medicine, School of Medicine, University of Virginia, Charlottesville, VA 22903, USA; 2 Division of Gastroenterology and Hepatology, Department of Medicine, School of Medicine, University of Virginia, Charlottesville, VA 22903, USA

**Keywords:** fecal microbiota transplant, *Clostridioides difficile*, refractory *C. difficile*, fulminant *C. difficile*

## Abstract

**Background:**

Options for *Clostridioides difficile* infection (CDI) refractory to conventional therapy are limited. Fecal microbiota transplant (FMT) is considered safe and effective treatment for recurrent CDI and could be a treatment option for refractory CDI. We investigated the efficacy and safety of FMT in hospitalized patients who were not responding to standard treatments for CDI.

**Methods:**

Electronic medical records of patients who received FMT inpatient for refractory CDI were reviewed as part of quality improvement efforts to evaluate safety and efficacy of FMT in inpatient setting.

**Results:**

Between July 2014 and December 2019, 9 patients (age 60–96) received FMT for CDI as inpatient for refractory or fulminant CDI. Most (7 of 9) of these patients had pseudomembranous colitis and underwent multiple FMTs (mean 2.15, range 1 to 3). Five patients had complete resolution and one patient had diarrhea that was *C. difficile*-negative. There was one recurrent CDI and two deaths, one of which may have been related to FMT or CDI. Compared to recurrent CDI at diagnosis, patients with refractory CDI had higher WBC and neutrophil counts, which decreased after FMT. The overall cure rate of FMT in refractory cases was 66.7%.

**Conclusions:**

This study shows moderate efficacy of FMT for treatment of refractory CDI although multiple FMT treatment may need to be administered in the presence of pseudomembranous colitis. Inpatient FMT may be an alternative strategy for managing refractory CDI in this population of patients who may not have any effective medical treatment available.

## INTRODUCTION

*Clostridioides difficile* is now the most common pathogen to cause healthcare-associated infections [1]. Prior to the COVID-19 pandemic, *C. difficile* infection (CDI) was the only infectious disease cause of death to increase in the United States in the last 20 years [2]. CDI is especially a big problem for the aging population. Even though patients older than 65 years of age make up about half of CDI patients, they make up around 90% of deaths from CDI [3]. Even after controlling for comorbidities, age has emerged as an important risk factor for severe disease and death [4]. Aging also affects response to therapy [5], making treatment of CDI challenging in the most vulnerable population. Among different types of CDI, those refractory to treatment with conventional CDI-directed antibiotic therapy pose the greatest challenge to treatment. IDSA (Infectious Diseases Society of America) clinical practice guidelines do not include any recommendations for refractory CDI, although fecal microbiota transplant (FMT) is among the potential therapeutic options mentioned [6]. Among the other therapeutic options, total abdominal colectomy is associated with high risk of adverse outcomes and significant morbidity as a result. Loop ileostomy with vancomycin lavage has been developed as an alternative with potential lower risk and morbidity [7] but there is also concern that the efficacy is lower [8]. Tigecycline or intravenous immunoglobulins are considered experimental, with little clinical data. FMT has emerged as a safe and effective treatment option for recurrent CDI, with multiple studies demonstrating strikingly superior efficacy of FMT to other treatment modalities for prevention of recurrences [9–11]. With increasing reports of favorable outcome from FMT, there have been efforts to expand the indications for use of FMT [12]. One such indication is for treatment of refractory or fulminant CDI with FMT. Recent studies, most notably from Hocquart et al. [13], but also in multiple observational studies, indicate that FMT is increasingly being used as treatment for refractory or fulminant CDI [14]. However, there are also concerns about risk for bacteremia or adverse outcomes with FMTs in critically ill patients [15], with inpatient status as one of the risk factors for worse outcomes. It is therefore of great interest to the clinician treating CDI to study the safety and efficacy of FMT for refractory CDI, especially in the context of older age. Our institution has adopted FMT as treatment for recurrent CDI in the outpatient setting in our Complicated *C. difficile* Clinic (CCDC) [16]. For patients admitted to the hospital, selected patients with CDI were treated with FMT, either for recurrent or refractory disease. The patients, with one exception, were older than 65 years of age. Patients gave informed consent to the therapy, which is still considered investigational by the FDA. We have collected clinical information from the patients who underwent FMT in the inpatient setting, to assess the efficacy and safety of the procedure.

## MATERIALS AND METHODS

### Patient Population

University of Virginia (UVA) Medical Center is a 619-bed tertiary care hospital in central Virginia. Patients received FMT while admitted to UVA Medical Center. CDI diagnosis was made by *tcdB* PCR (Cepheid, Sunnyvale, CA, USA). There were two types of patients referred for evaluation: patients with multiple recurrent CDI episodes and patients with CDI refractory to conventional treatment or fulminant CDI. Recurrent CDI, as defined in IDSA guidelines, is defined as an episode of symptom onset and positive *C. difficile* test result following CDI in the previous 2–8 weeks [6]. The criteria for FMT in recurrent CDI is the same as that used in our outpatient Complicated *C. difficile* Clinic (CCDC) [16]. Briefly, patients were eligible for FMT if they had three or more recurrences of CDI despite appropriate treatments for previous episodes including at least one vancomycin taper [16]. Excluded patients included patients with neutropenia (absolute neutrophil count (ANC) < 500), patients who had undergone solid organ or bone marrow transplantation within the prior year, or patients whose primary oncologic or transplant team were not in agreement with FMT. IDSA guidelines do not provide a definition of refractory CDI [6]. In our center, refractory CDI was defined as an active episode of CDI that did not respond to conventional therapy, including metronidazole, vancomycin, and/or colectomy/ileostomy. Fulminant CDI, per IDSA guidelines, were CDI episode characterized by hypotension or shock, ileus, or megacolon [6]. These patients were referred to Infectious Diseases, Gastroenterology, or Surgery for consultation regarding potential FMT. FMT was performed if there was consensus among the specialists that benefits outweighed the risks of the FMT. Patients signed informed consent acknowledging the fact that the procedures were considered investigational use in CDI not responding to standard treatment as per 2014 FDA guidance.

### Study Design

We performed a retrospective chart review of patients who underwent FMT from July 2014 to December 2019 to evaluate the safety and efficacy of FMT in the inpatient setting for refractory CDI as part of the efforts by the Antimicrobial Stewardship Program and the Antibiotic Utilization Committee. We reviewed the electronic medical record of each patient to collect patient demographics, CDI history, laboratory tests results, and clinical data from encounter notes. As this research involves secondary use of identifiable private information which is for the purpose of conducting quality assessment and improvement activities, including outcomes evaluation and development of clinical guidelines, is exempt from IRB approval.

### FMT Procedure

FMT procedure was similar as the procedure described in Shin et al. [16] for outpatient FMT in the Complicated *C. difficile* Clinic. Briefly, universal donor specimens were prescreened and purchased from OpenBiome (Boston, MA, USA). These stool samples were stored frozen and delivered to the inpatient unit using Pyxis system used in UVA Medical Center for medication dispensation. FMT was mainly performed through colonoscopy, but if the patient had special circumstances other routes were utilized, such as ileoscopy or upper gastrointestinal feeding tube in patients with ileostomy or colectomy. If pseudomembranes were visualized on colonoscopy during FMT, repeat FMTs were performed until there were no pseudomembranes seen on colonoscopy, similar to the protocol proposed by Fischer et al. [12] After FMT, there were no set protocol regarding treatment with *C. difficile* active antibiotics and decision to resume antibiotics was left to the discretion of the physician treating the patient. Eight out of the nine patients treated for refractory CDI received antibiotics after FMT.

### Study Outcomes

The patient’s clinical progression was followed by reviewing the electronic medical record of the patient’s concurrent hospitalization. Outcomes were noted as resolved, recurrence, deaths, or *C. difficile*-negative persistent diarrhea. Stool *C. difficile* toxin B gene PCR was performed for diagnosis of infection. Recurrence was diagnosed if there was return of symptoms after resolution and positive *C. difficile* PCR from stool during this recurrent episode. The recurrence, deaths, and persistent diarrheas were documented if they occurred within 90 days of the first FMT.

### Statistical Analysis

Student’s *t* test or Mann-Whitney *U* test were used for analysis. A 2-tailed *P* value of 0.05 was considered statistically significant (GraphPad Prism (La Jolla, CA, USA)).

## RESULTS

### Demographics

**Figure 1.**
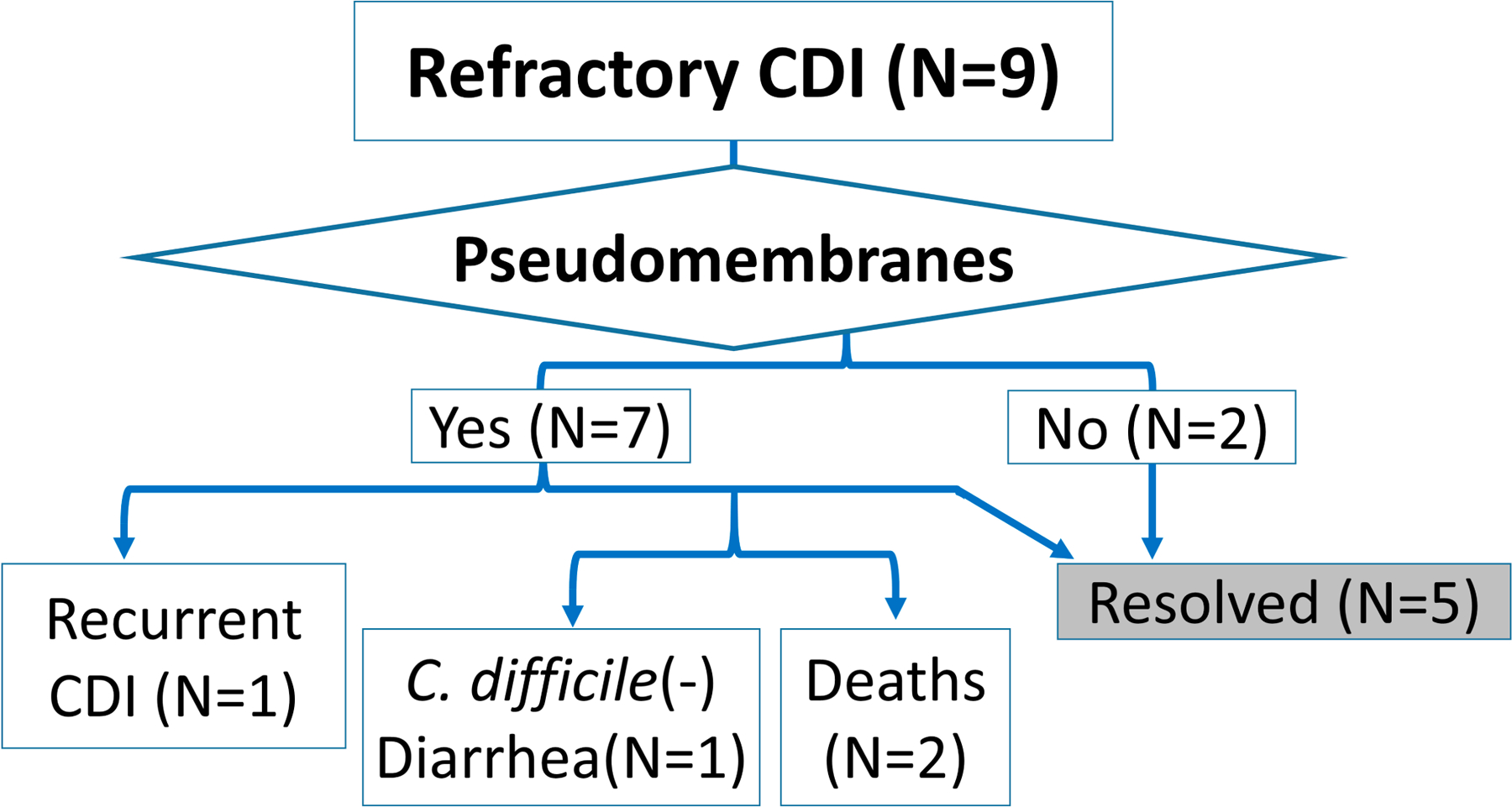
Outcomes of patients who received FMT for recurrent or refractory CDI.

Starting in July of 2014, 13 patients received fecal microbiota transplant (FMT) for CDI in the inpatient setting at the University of Virginia Health System. There were two different patient populations who received FMT in the inpatient setting. One group of patients (four out of the thirteen total inpatient FMT patients) were eligible for FMT per protocol in the Complicated *C. difficile* Clinic (CCDC) due to presence of 3 or more recurrent episodes of CDI. A second group of patients (nine out of the thirteen total inpatient FMT patients) received FMT due to the lack of efficacy of conventional CDI-directed antibiotic therapy (oral or rectal vancomycin plus intravenous metronidazole). Their mean age was 76 years and 5 out of 9 were male. All nine patients had severe CDI per IDSA guidelines. The details of the individual cases are described in Table 1 and outcomes are presented in Figure 1.

### Clinical Course

**Figure 2.**
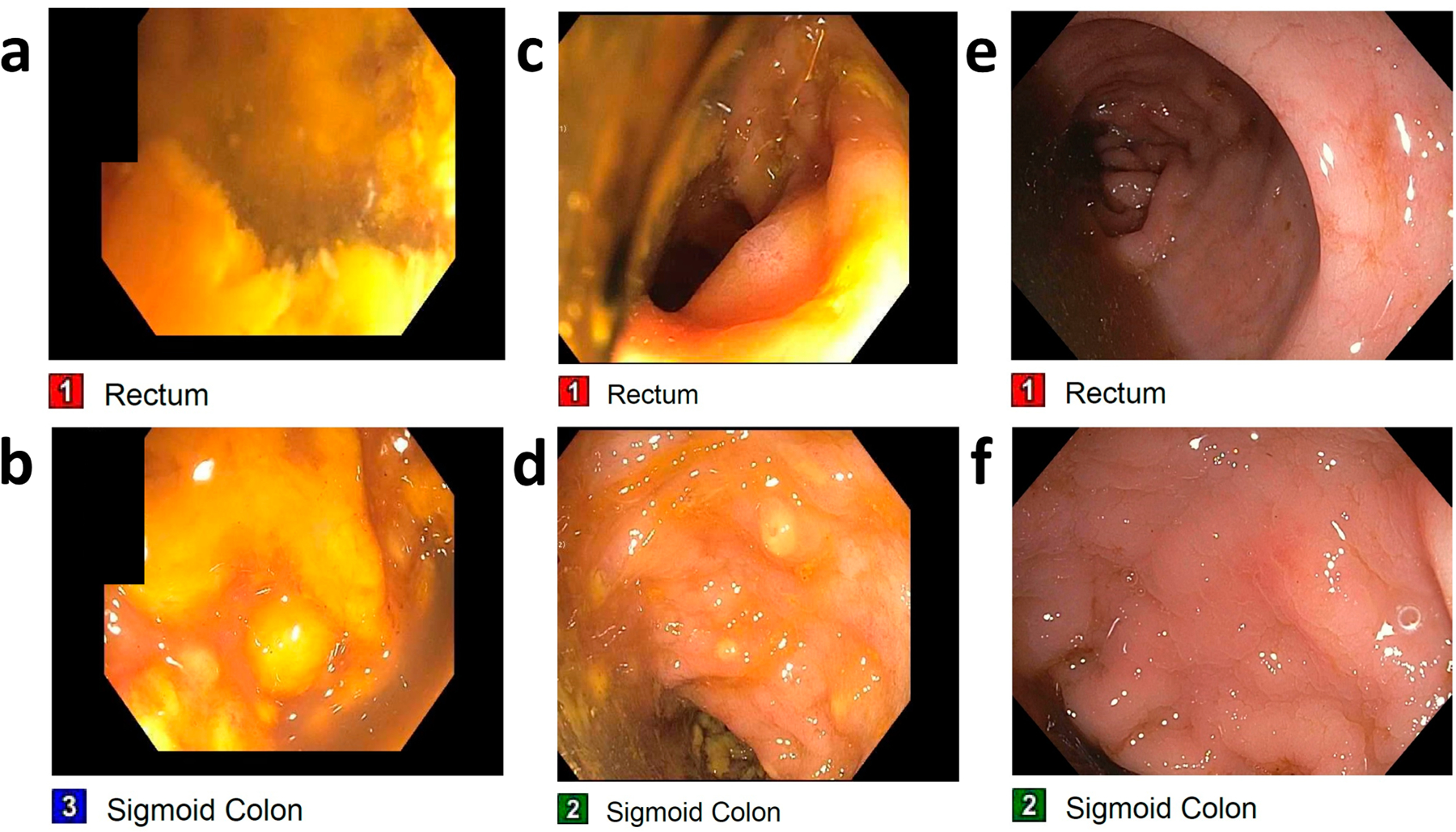
Colonscopic changes after FMT in patients with pseudomembranes from CDI. Colonoscopic findings from one patient shows yellow pseudomembranes covering both the rectum (**a**) and the sigmoid colon (**b**) during the first FMT. There is some improvement on the second FMT in the rectum (**c**) and the sigmoid colon (**d**). On the patient’s third FMT, both the rectum (**e**) and the sigmoid colon (**f**) exhibited resolution of pseudomembranes and healthy intestinal epithelium.

According to the protocol by Fischer et al. [12], all of the patients who had pseudomembranes on colonoscopy had repeat FMT every 3–5 days until resolution of pseudomembranes. Regarding post-FMT care, Fischer et al. [12] recommends treatment with PO vancomycin. In our patients we did not have a set protocol for antibiotic administration, and all but one patient received antibiotics. Among the nine patients who received FMT for refractory or fulminant CDI, seven (78%) patients had pseudomembranes visualized on colonoscopy (Figure 2). The average number of FMTs performed in all refractory CDI patients is 2.15 (range 1–3). One of the two patients without pseudomembranous colitis had persistent diarrhea after initial FMT and had a repeat FMT after 12 days, which showed pseudomembranes, necessitating a third FMT in another 7 days. After the 3 FMTs, the diarrhea resolved. The second patient without pseudomembranes had prompt response to FMT and was discharged from the hospital, but had recurrence of diarrhea symptoms with positive *C. difficile* and was treated with another FMT 35 days after the first FMT, with resolution of the diarrhea. Among the seven patients with pseudomembranes, one patient withdrew care due to the terminal nature of the kidney disease as patient had advance directives specifying no dialysis treatments, and passed away after one FMT. Three of the seven patients received three FMTs following protocol, and had resolution of diarrhea. The rest of patients with pseudomembranes (three out of nine patients) received two FMTs. Two patients had pseudomembranes visualized on colonoscopy but did not have a third FMT and resulted in one case of recurrent CDI and one case of persistent *C. difficile*-negative diarrhea, both of which were treated with fidaxomicin. One patient had severe sepsis at the time of FMT and died soon after. Overall, there were three failures, one recurrence and two deaths (regardless of causes), leading to a 33.3% failure rate, or 66.7% success rate for FMT performed for refractory cases.

### Deaths

There were two patients who died within 90 days. The first patient was an 87 year old woman with history of HTN and CKD stage 4 who was admitted for CDI refractory to treatment, which was precipitated by antibiotic therapy for diverticulitis. Patient received FMT via flexible sigmoidoscopy one day after FMT as part of the treatment plan recommended by gastroenterology. Patient responded to FMT with a decrease in stool frequency but due to patient’s wishes to not receive dialysis, patient passed away from kidney failure 4 days after FMT. The second patient was a 73 year old man with prostate cancer s/p resection, myeloproliferative disorder, pulmonary embolism treated with apixaban for 3 months, and Coombs-positive hemolytic anemia on chronic steroids. Patient was initially admitted to the hospital with fever, rash, eosinophilia, lactic acidosis and respiratory failure. The diagnosis was unclear, with sepsis, strongyloidosis with potential for hyper-infection syndrome, drug reaction with eosinophilia syndrome (DRESS), and hematologic malignancies all considered possible causes. Patient was treated with antihelminthics for parasitic infections, multiple broad spectrum antibiotics for bacterial or fungal infections, and immune suppression for DRESS. 2 weeks after admission, patient developed fever, diarrhea, and CT abdomen/pelvis finding of diffuse wall thickening, surrounding edema, and mild dilation of the colon consistent with severe infectious colitis. Patient immediately underwent loop ileostomy and vancomycin lavage but developed worsening leukocytosis and persistent high volume output through ileostomy. FMT was performed 10 days after loop ileostomy via ileoscopy while continuing treatment with IV metronidazole and PO vancomycin then fidaxomicin. Patient was not evaluated for pseudomembranes. Patient initially improved and was able to transfer to the floors from intensive care unit (ICU), but had new leukocytosis and fever after 2 weeks, leading to ICU admission again. Patient received a second FMT about 18 days after the first FMT with no pseudomembranes noted via colonoscopy. Patient continued to decline with sepsis and patient passed away after withdrawing care. Interestingly, patient also developed worsening rash with peripheral eosinophilia and atypical lymphocytes as well as a high CMV viral load for which he was started on IV ganciclovir therapy for possible cytomegalovirus (CMV) colitis.

### Blood Counts and Biochemistry after FMT

**Figure 3.**
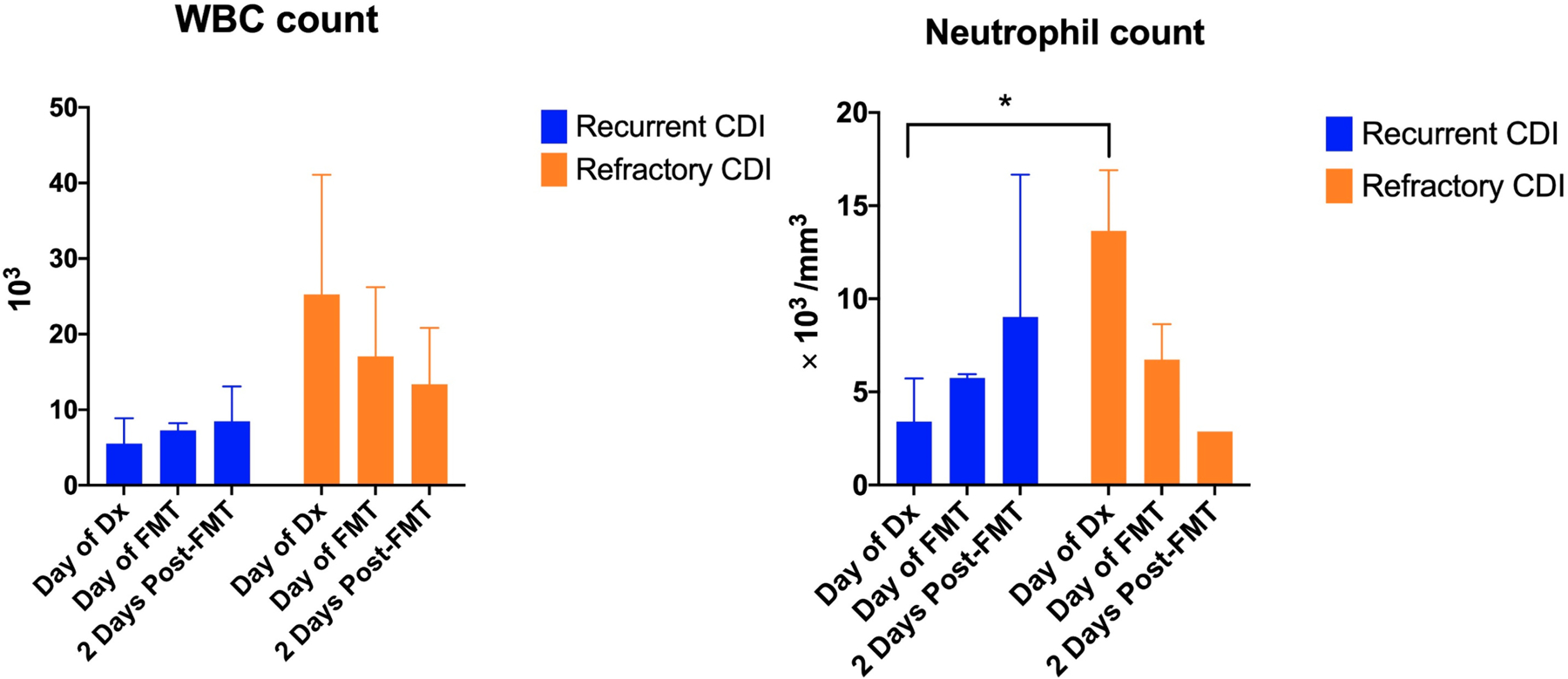
Leukocyte response to CDI and FMT WBC count was higher at diagnosis in the patients with refractory CDI compared to patients with recurrent CDI, although without statistical significance. The WBC count had a trend toward decreasing with FMT in refractory CDI. Neutrophil count was statistically significantly higher at diagnosis in refractory CDI compared to recurrent CDI and had a trend towards decrease with FMT.

When compared to the recurrent CDI group (5.523 ± 3.353), WBC count in the refractory CDI group was higher (25.25 ± 15.84) at the time of diagnosis, but without statistical significance (Figure 3). The WBC count did not change significantly with FMT, but there was a trend towards decreasing with FMT in the refractory CDI group. Neutrophil count in refractory CDI (13.64 ± 3.262) was higher at the time of diagnosis than in the recurrent group (3.403 ± 2.312) (*p* < 0.05). The change with FMT was not statistically significant, but there was a decrease in neutrophil count with FMT in refractory or fulminant group while an increase in neutrophil count in the recurrent group. CRP levels were checked in only 4 patients. Lactic acid level difference was not statistically significant but there was a trend toward a higher level in refractory CDI (3.017 ± 0090) than recurrent CDI (1.250 ± 0.2121) at the time of diagnosis. Albumin and creatinine levels were not different between refractory and recurrent CDI and there were not significant changes with FMT.

## DISCUSSION

FMT has become the most effective treatment for recurrent CDI, with a cure rate around 80–90% [9,11]. As noted earlier, there is no consensus for best treatment options for CDI refractory to conventional therapy [6]. Previous studies have included refractory with the recurrent CDI cases and thus, did not evaluate refractory cases separately [17–20] except in one case series from South Korea looking at the response of patients with refractory CDI to FMT [21] and an observational study evaluating the effect of adapting FMT as institutional practice on overall institutional mortality of patients with refractory CDI [22]. One difficulty is the definition of “refractory” as opposed to “recurrent”. Some studies define “refractory” as “recurrent CDI after multiple treatment courses [18,19]”, which is more consistent with “recurrence”. Fulminant CDI is similarly difficult to study, because studies evaluating fulminant CDI usually defines the patient populations as “severe and fulminant”, including severe CDI (WBC count of ≥15,000 cells/mL or a serum creatinine of >1.5 mg/dL) as defined by IDSA guidelines, which can be quite broad [23]. The lack of accuracy in reporting or definition of cases makes the interpretation of data from FMTs studies even more difficult. In our study, we were able to separate the cases by the indication for FMT. There were 7 of 9 (66.7%) patients with refractory disease who responded to FMT, which is somewhat moderate compared to the very high success rates seen in recurrent CDI [9,11], but since there is no standard of treatment and expected cure rate is unknown, this is still a promising result and warrant further investigation.

An additional factor to note is that our patient population were all advanced in age, being older than 60, with mean age of 76. A study evaluating the performance of FMT for recurrent CDI in older adults also resulted in a much lower success rate (67.2%) [24] than expected (~90%) [9,11]. As of now, it is unknown if the age of the recipient of FMT determines the outcome, but an expanded study including adults of all ages my shed new light on the interaction of age and FMT response.

The protocol for FMT was designed similarly to Fischer et al., which involves repeat FMT when pseudomembranes are present, with additional 5 days of PO vancomycin administered between the repeat FMTs [12]. The effectiveness of repeat FMTs in patients with pseudomembranes was seen in multiple studies [10,12,14]. Addition of antibiotics in between FMTs have been used with some success in small observational studies [12,14], although the effect of the antibiotics on the microbiota after transplant is not known.

WBC count is a well-known marker of CDI severity [6], but neutrophil count is another factor that is strongly associated with severe outcome/deaths [25]. The elevated WBC count and neutrophil count seen at diagnosis with refractory CDI compared to recurrent CDI suggests higher inflammatory state with more severe disease. The decrease of these measures with FMT may suggest that FMT has an immunomodulatory effect.

Regarding the safety, there is concern about the risk of administering live bacterial culture to a patient with potential disruption in mucosal integrity [26]. The recent report of multidrug-resistant *E. coli* bacteremia transmitted by FMT [27] further questions the safety of the procedure. In our study, two deaths occurred within 90 days of the procedure in the patients who underwent FMT. One death attributed to renal failure which predated the CDI and another patient who died from potential sepsis. The diagnosis for the latter case was not completely clear, because patient originally presented to the hospital prior to CDI diagnosis with unexplained eosinophilia pointing to a potential parasite infection, DRESS, or hematologic malignancy, and the diagnosis was still unclear at the time of death, making the association with FMT not compelling. However, one notable issue with the patient was the CMV viremia. Patient had elevated CMV viral load after FMT and was treated with IV ganciclovir prior to patient’s death. Interestingly, there is literature suggesting the possibility of CMV reactivation in solid organ transplant recipients undergoing FMT [15]. In this study, it was noted that there was a higher rate of reactivation of CMV in patients who were seropositive prior to the FMT [15]. Unfortunately, the patient was not screened for CMV status prior to viremia, so previous serologic status cannot be determined. Unlike in cases of solid organ transplants or bone marrow transplants, it is unknown at the present what role the CMV seropositive/seronegative status of donors and recipients plays in the outcome in fecal transplants. With the findings in this study in addition to the literature in mind, CMV status in donors and recipients in FMT should be studied. Also, regarding the risk of systemic dissemination of intestinal bacteria, none of the patients treated with FMT developed bloodstream infections, including the deaths. This is consistent with the recently published study demonstrating that FMT actually results in lower incidence of bloodstream infections than conventional antibiotic therapy [28].

It was surprising that the number of FMTs performed over 6 years were 13 in a large tertiary care university hospital. As the decision to choose certain therapeutic options is the result of multiple factors, including patient population, institutional oversight, and individual physician expertise and preference, no conclusions can be drawn from this observation. However, it is not news that there is still significant concern about the safety and efficacy of FMT [26]. Although as clinicians we must follow the rule of “do no harm”, the benefits and risks must be weighed, especially in cases with limited treatment options. The results from this study are quite promising for utilizing FMT in refractory CDI cases even among the elderly, and should be considered for a larger prospective study.

## Figures and Tables

**Table 1.** Details of patients who received FMT for CDI.

Patient characteristics	# of CDI prior to FMT	Precipitating event	Colonoscopic finding	Response to first FMT	Antibiotics post-FMT
87 year old woman with hypertension (HTN), chronic kidney disease (CKD) stage 4	1	Antibiotic therapy for diverticulitis	Pseudomembranes	Died due to stopping dialysis per patient wishes	Intravenous metronidazole
75 year old woman with Diabetes mellitus (DM), HTN, hyperlipidemia, depression, anxiety	1	Multiple antibiotics for sepsis	Pseudomembranes	Slight improvement, but FMT as planned due to pseudomembrane	Rectal vancomycin
60 year old woman with Coronary artery disease s/p stent, HTN, CDI s/p colectomy and ileostomy in with take down	2	Ileostomy takedown (diarrhea started 10 days after surgery)	Pseudomembranes	Improved, but 2nd FMT performed according to original plan	Oral vancomycin
96 year old man with CAD s/p coronary artery bypass graft, severe aortic stenosis s/p replacement, atrial fibrillation on apixaban, deep vein thrombosis, CDI with toxic megacolon in s/p loop ileostomy s/p take down	2	Ileostomy takedown?	Pseudomembranes	Persistent diarrhea	Oral vancomycin
65 year old man with bilateral lower extremity lymphedema with chronic wounds c/b osteomyelitis and cellulitis, monoclonal gammopathy, meningioma	4	PPI use	No pseudomembranes	Recurrence after 4 days, repeat FMT	Oral vancomycin
72 year old woman with colon cancer, DM, gastroesophageal reflux, HTN, asthma	1	Laparoscopic extended right colectomy	Pseudomembranes	Persistent, repeat FMT	Oral fidaxomicin
73 year old man with prostate cancer s/p resection, myeloproliferative disorder, pulmonary embolism s/p 3 months apixaban, Coombs-positive hemolytic anemia on steroid	1	Multiple antibiotics for sepsis	Pseudomembranes	Persistent, worsening sepsis, repeat FMT	Intravenous metronidazole + oral fidaxomicin
72 year old man with CAD s/p percutaneous coronary intervention, congestive heart failure, HTN, DM, recurrent infection, recurrent CDI	4	Prolonged stay at LTACH	Pseudo-membranes	Improved, but 2nd FMT given according to protocol	Intravenous metronidazole + oral fidaxomicin
82 year old man with HTN	1	Multiple antibiotics for dental infection	No pseudo-membranes	Improved, but had recurrent diarrhea and was given 2nd FMT with resolution of diarrhea	None

## Data Availability

The dataset from the study is not available because of confidentiality of health information.

## ABBREVIATIONS

CDI: *Clostridioides difficile* infection
FMT: fecal microbiota transplant
ANC: absolute neutrophil count
